# Active Learning Norwegian Preschool(er)s (ACTNOW) – Design of a Cluster Randomized Controlled Trial of Staff Professional Development to Promote Physical Activity, Motor Skills, and Cognition in Preschoolers

**DOI:** 10.3389/fpsyg.2020.01382

**Published:** 2020-07-03

**Authors:** Eivind Aadland, Hege Eikeland Tjomsland, Kjersti Johannessen, Ada Kristine Ofrim Nilsen, Geir Kåre Resaland, Øyvind Glosvik, Osvald Lykkebø, Rasmus Stokke, Lars Bo Andersen, Sigmund Alfred Anderssen, Karin Allor Pfeiffer, Phillip D. Tomporowski, Ingunn Størksen, John B. Bartholomew, Yngvar Ommundsen, Steven James Howard, Anthony D. Okely, Katrine Nyvoll Aadland

**Affiliations:** ^1^Department of Sport, Food and Natural Sciences, Faculty of Education, Arts and Sports, Western Norway University of Applied Sciences, Sogndal, Norway; ^2^Center for Physically Active Learning, Faculty of Education, Arts and Sports, Western Norway University of Applied Sciences, Sogndal, Norway; ^3^Department of Pedagogy, Religion and Social Studies, Faculty of Education, Arts and Sports, Western Norway University of Applied Sciences, Sogndal, Norway; ^4^Department of Strategic Initiatives, Faculty of Education, Arts and Sports, Western Norway University of Applied Sciences, Sogndal, Norway; ^5^Department of Sports Medicine, Norwegian School of Sport Sciences, Oslo, Norway; ^6^Department of Kinesiology, Michigan State University, East Lansing, MI, United States; ^7^Department of Kinesiology, University of Georgia, Athens, GA, United States; ^8^Norwegian Centre for Learning Environment and Behavioural Research in Education, University of Stavanger, Stavanger, Norway; ^9^Department of Kinesiology and Health Education, The University of Texas at Austin, Austin, TX, United States; ^10^Department of Coaching and Psychology, Norwegian School of Sport Sciences, Oslo, Norway; ^11^Early Start and School of Education, University of Wollongong, Wollongong, NSW, Australia; ^12^Illawarra Health and Medical Research Institute, Wollongong, NSW, Australia

**Keywords:** preschool, public health, professional development, enriched physical activity, child development, motor competence, cognition, integration

## Abstract

**Introduction:**

There is a dearth of high-quality evidence on effective, sustainable, and scalable interventions to increase physical activity (PA) and concomitant outcomes in preschoolers. Specifically, there is a need to better understand how the preschool context can be used to increase various types of physically active play to promote holistic child development. The implementation of such interventions requires highly competent preschool staffs, however, the competence in promoting PA is often low. The main aim of the ACTNOW study is therefore to investigate the effects of professional development for preschool staffs on child PA and developmental outcomes.

**Methods:**

The study will be conducted in Norway 2019–2022 and is designed as a two-arm (intervention, control) cluster randomized controlled trial (RCT) with 7- and 18-months follow-ups. We aim to recruit 60 preschools and 1,200 3- to 5-years-old children to provide sufficient power to detect effect sizes (ESs) between 0.20 and 0.30. The intervention is nested within two levels: the preschool and the child. Central to the ACTNOW intervention are opportunities for children to engage in a variety of “enriched,” meaningful, and enjoyable physically active play that supports the development of the whole child. To this end, the main intervention is a 7-month professional development/education module for preschool staff, aimed to provide them with the necessary capacity to deliver four core PA components to the children (moderate-to-vigorous PA, motor-challenging PA, cognitively engaging play, and physically active learning). We will include a range of child-level outcomes, including PA, physical fitness, adiposity, motor skills, socioemotional health, self-regulation, executive function, and learning. At the preschool level, we will describe implementation and adaptation processes using quantitative and qualitative data.

**Discussion:**

Professional development of staff and a whole-child approach that integrates PA with cognitively engaging play and learning activities in the preschool setting may provide a feasible vehicle to enhance both physical and cognitive development in young children. ACTNOW is designed to test this hypothesis to provide a sustainable way to build human capital and provide an early solution to lifelong public health and developmental challenges.

**Clinical Trial Registration:**

www.ClinicalTrials.gov identifier NCT04048967.

## Introduction

Giving every child the best start in life should be the highest priority to address social inequalities and to improve population health, laying the foundation for equitable development of human capital and life opportunities (Ministry of Health and Care Services, 2007; Marmot, 2010). Physical activity (PA) provides young children opportunities for developing their physical, motor, socioemotional, and cognitive skills. PA plays a key role in preventing non-communicable diseases and at an early age provides a foundation for optimal health and development that represents a critical investment in children’s development of human capital (Bailey et al., 2013; Carson et al., 2017). Yet, there are growing concerns regarding low PA levels among children and youth, including preschoolers (Bornstein et al., 2011; Driediger et al., 2018; Truelove et al., 2018; Nilsen et al., 2019c). The preschool (97% coverage among 3- to 5-years-old in Norway) and school settings are optimally suited to reach most children, irrespective of social background, with initiatives to increase PA levels and improve health and development. Since interventions before school age provide the most cost-effective solutions (Doyle et al., 2009), there is a need for broad, scalable interventions to increase PA in preschoolers. However, the existing interventions have lacked effectiveness (Finch et al., 2016; Wick et al., 2017), and only a few studies with suboptimal designs have investigated cognitive outcomes (Carson et al., 2016). As a result, evidence is insufficient to shape policy. *In response, the ACTNOW study has two main research rationales. First, to address the lack of evidence of effective and sustainable interventions to increase PA and concomitant outcomes in preschoolers, including evidence on how, why, and for whom interventions may work or not. Second, to provide evidence about the effect of PA on young children’s mental health and cognitive development, including measures of socioemotional health, self-regulation, executive function, and learning.*

Overwhelming evidence shows that PA promotes physical health and reduces the risk of non-communicable diseases in adults (Pedersen and Saltin, 2015) and risk factors for non-communicable diseases in children (Poitras et al., 2016). There is also increasing evidence that PA beneficially affects brain health, cognition, and learning in children (Donnelly et al., 2016; Alvarez-Bueno et al., 2017a, b; Norris et al., 2019). Recent systematic reviews have shown positive effects of PA on cognitive outcomes in 3- to 18-years-old, but effects are generally small and inconclusive (mean standardized ESs 0.11-0.30 across cognition and academic outcomes) (Alvarez-Bueno et al., 2017a, b; Singh et al., 2019). However, cognition and learning outcomes are rarely reported in children under 6 years of age (Norris et al., 2019; Singh et al., 2019), a period during which brain development is greatest (Thompson and Nelson, 2001) and the brain is most susceptible to stimuli. In addition, the existing studies are methodologically weak (Carson et al., 2016; Norris et al., 2019; Singh et al., 2019). Consider a recent systematic review in 0- to 5-years-old (Carson et al., 2016); of the five experimental studies included, no studies were rated high quality, no studies investigating chronic effects used a randomized controlled design, and sample sizes were low (10-94 participants). Thus, there is an urgent need for high-quality, rigorous research, with large samples, that examines how PA affects cognitive development in young children (Norris et al., 2019; Singh et al., 2019).

Several causal mechanisms have been proposed to explain how PA affects children’s cognitive development through altered brain structure and function (Best, 2010; Meeusen et al., 2018). Mechanisms may be impacted by the quantity or dose (intensity, duration, and/or frequency) of PA (Howie et al., 2015; Howie and Pate, 2018) and by the type or quality of PA due to the cognitive or coordinative demands inherent in PA (Pesce, 2012; Diamond, 2015; Pesce et al., 2018). Qualitative features may relate to motor challenges (adapting to new or complex movement patterns) (Diamond, 2000; Guadagnoli and Lee, 2004; van der Fels et al., 2015), the complexity of the PA context (structured games or active play demanding executive functions) (Chang et al., 2013; Schmidt et al., 2015; Tomporowski et al., 2015), and/or PA integrated with other learning activities (physically active learning) (Mullender-Wijnsma et al., 2016; Mavilidi et al., 2017, 2018a). Evidence from the latter line of research, focusing on the qualitative features of PA, points to the importance of children’s mindful engagement in the PA to optimally stimulate their socioemotional and cognitive development and learning. Within this line of research, PA should be “enriched” to provide children with physical activities that are cognitively challenging, emotionally loaded, and socially engaging (Pesce et al., 2018). Enrichment of PA through manipulation of the task and/or environment requires goal-directed behavior underpinned by higher-order cognitive control mechanisms or executive functions (Best, 2010). The joint focus on motor competence and cognition (Pesce, 2012; Diamond, 2015; Pesce et al., 2018) bridges research targeting the effect of PA for both physical and mental developmental outcomes. These perspectives substantially broaden the traditional focus on PA: moving away from the predominant attention to the intensity and metabolic demands of PA to also include the use of mindful PA. Given this framework, more high-quality research is needed to investigate how these foci and features of various activities can be integrated to jointly impact children’s physical fitness, motor competence, executive function, socioemotional development, self-regulation, and learning.

Studies of interventions to increase objectively measured PA (Finch et al., 2016) and motor competence (Wick et al., 2017) in children aged 0-6 years have shown small to moderate effects. These are disappointing and driven, in part, by poor study quality (Wick et al., 2017) and the challenge of translating well-controlled efficacy trials to effectiveness studies in preschool settings. Separate meta-analyses by Finch et al. (2016, 15 studies investigating PA) and Wick et al. (2017, 26 studies investigating motor competence) revealed that efficacy trials have the potential to significantly improve PA and motor competence in young children, whereas effectiveness trials – performed under real-world conditions – are not beneficial. Specifically, short-term interventions and interventions delivered by external “experts” were effective (mean standardized ESs 0.58-1.54), whereas long-term interventions and interventions delivered by preschool staff were ineffective (ESs 0.07-0.41). Similar effects are shown for cognitive outcomes across studies with preschool- and school-aged children (Norris et al., 2019). These findings show that the benefit of PA is tangible, but there exist no effective, scalable solutions to increase PA in the actual preschool setting, which provides limited direction for policymakers and practitioners.

Promoting PA in an enjoyable and motivating manner, with a focus on moderate-to-vigorous PA (MVPA) that stimulates motor competence and integrates with other preschool activities and objectives, requires great effort and thus competent and motivated preschool directors and staff. However, previous preschool studies have provided minimal teacher training and support. Among the five high-quality studies reviewed by Wick et al. (2017) where the intervention was delivered by the staff, and among the five relevant studies deemed pragmatic in the review by Finch et al. (2016), studies provided only one to five workshops along with some follow-up sessions. More recent studies, still showing equivocal effects, offer similar low amounts of teacher training (Goldfield et al., 2016; Jones et al., 2016; Adamo et al., 2017; Tucker et al., 2017). These are contrasted with examples such as the *Study of Health and Activity in Preschool Environments* (SHAPES, United States), *Motor Skills in PreSchool* (MiPS, Denmark), and *Joy of Moving* (Italy), all of which established stronger relationships between interventionists and staff through teacher training, workshops, and follow-ups than most other studies (Pfeiffer et al., 2013; Howie et al., 2014; Pesce et al., 2016; Hestbaek et al., 2017). The lack of staff professional development is concerning because there is a well-known challenge of a low competence level in the preschool sector internationally. In Norway, <50% of the preschool staff are certified teachers (The Norwegian Directorate of Education, 2016), and there is a specific lack of staff’s competence in promoting PA, and probably more so in promoting PA aimed to affect motor skills, cognition, and learning. As a result, we argue that much of the failure to support preschool interventions is due to the lack of director and staff training. Moreover, well-trained staff can become partners in the co-creation of the specific components of the intervention as applied to their preschool, along with ongoing efforts to evaluate and continuously improve the intervention. That is, not only must the staff be sufficiently trained to implement the program, they must be sufficiently trained to modify and improve on the intervention to support long-term sustainability. Finally, randomized controlled trials (RCTs) are unable to explain why an intervention may work in a particular context or for a particular group of participants. Thus, examining the ways an intervention is put into practice and delivered to its participants is fundamental to avoid an inaccurate attribution of the cause(s) of results (Lendrum and Humphrey, 2012; Durlak, 2016; Humphrey et al., 2018). It is well documented that implementing interventions into the school setting is complex and challenging for a variety of reasons (Lendrum and Humphrey, 2012), but less is known about the preschool setting. Thus, a thorough description of the implementation and evaluation of these processes should be included in future trials as a prerequisite to scaling interventions in the real world (Howie et al., 2014; Reis et al., 2016).

### Research Gap and Research Questions

There is an urgent need for large, high-quality PA intervention studies in preschool that can demonstrate effectiveness in a dynamic and complex real-world setting and that are scalable to broader national and international contexts. Therefore, the aim of this study is to investigate the effects of an education module, which is designed to create preschool staff who are highly competent in promoting various aspects of physically active play that, in combination, have the potential to simultaneously affect various developmental and learning outcomes, underpinning a whole-child approach. If effective, ACTNOW will provide further evidence of the efficiency, acceptability, and feasibility of the preschool setting in building human capital and providing an early solution to lifelong public health and developmental challenges.

Two main research questions will be tested using both quantitative and qualitative methods, applied to both the child and preschool levels:

1. How does the intervention affect children’s PA, physical fitness, motor competence, socioemotional health, self-regulation, executive functions, and learning?
2. How does the intervention interact with different preschool contexts to produce various individual and organizational outcomes?

## Methods and Analysis

### Design

The research questions will be investigated using a two-arm (intervention; control) cluster RCT with randomization at the preschool level, including 7- and 18-months follow-ups. Consistent with recommendations from implementation research in the school setting (Lendrum and Humphrey, 2012; Durlak, 2016), ACTNOW will include strong involvement from preschool owners and staff to provide broad support and anchor the project in the preschool sector. Thus, we aim to combine a large-scale experimental study with intervention co-creation and a continuous improvement effort in this sector. We will achieve this combination by making a compromise between the standardization needed in an experimental trial and the adaptations and flexibility needed for the intervention to be accepted, internalized, and further developed by the preschools. This approach is consistent with favorable effects of user adaptation of interventions (Søvik et al., 2017) and will hopefully improve staff participation in the professional development and thus effectiveness of the intervention over previous similar interventions (Finch et al., 2016; Pesce et al., 2016; Wick et al., 2017). This approach overcomes a common criticism of clinical trials as it increases the value for later scaling and dissemination to the real-world settings. In this way, we aim to create sustainable solutions for improved child development and public health. Thus, the intervention’s development, implementation, and evaluation are framed within a “realist RCT” approach (Bonell et al., 2012) providing the rationale for a thorough process evaluation of the study.

ACTNOW is registered in the ClinicalTrials.gov database with identification number NCT04048967 (August 7 2019)^1^ prior to recruiting any participants.

### Intervention and Theoretical Framework

**FIGURE 1:**
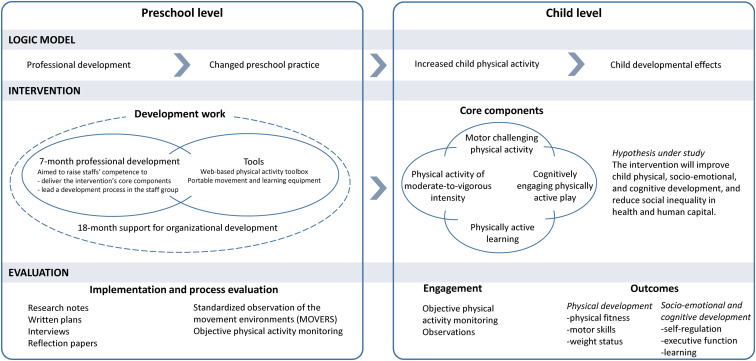
The two-level intervention model.

The intervention is framed within a socioecological model (McLeroy et al., 1988), placing the preschool as an influential factor for children’s health and development. The intervention is nested within two levels (Figure 1); the *preschool* and the *child*, of which will be implemented concurrently. On the child level, the intervention is composed of four core components of various types of PA. Given this complexity and the challenge of low staff qualifications to implement PA, the 7-month professional development *Active Learning Preschoolers* will be offered to preschool directors and teachers during Year 1. The professional development is structured as a 15-credit continuing education module that provide staff the opportunity to achieve credits for their efforts. Moreover, the preschools will be equipped with tools to support the intervention implementation, including a web-based PA toolbox (an ideas bank of child activities framed within the four core intervention components) and portable movement and learning equipment, providing a basis for implementing the new PAs. Finally, preschools will be offered continuous support over the 18-month intervention period to facilitate the intervention implementation (Years 1 and 2). The study is based on the logic model that the professional development, additional resources, and support will change preschool practices, which will in turn increase and improve children’s PA opportunities that will cause child developmental effects. Thus, the aim of the preschool level intervention is to provide preschool staff the necessary expertise, resources, and capacity to intervene on the child level. Researchers will not directly deliver the intervention at the child level.

At both the *preschool* and the *child* levels, we seek to create a mastery-motivational climate as well as an autonomy supportive climate guided by the Achievement Goal Theory (AGT) (Ames, 1992) and Self-Determination Theory (SDT) (Ryan and Deci, 2000). Together, these two perspectives are hypothesized to provide social-contextual conditions expected to facilitate learning, growth, and development among preschool directors, teachers, as well as the children. A mastery climate emphasizes effort, progress, and optimal challenges facilitating the intrinsic value of learning. According to AGT, creating a mastery climate will help influence a person’s beliefs, attributions, affect, and goal of action. Specifically, creating a mastery-oriented climate will help individuals to relate effort to mastery and success, to take interest in developing new skills and improve their level of competence, and evaluate their achievement by using self-referenced standards (Ames, 1992). According to the SDT perspective, an autonomy supportive climate, as well as a structure supportive and a relatedness supportive climate, will provide social-contextual conditions that help stimulate intrinsic motivation, self-regulation, and socioemotional health through three basic psychological needs: competence, relatedness, and autonomy (Ryan and Deci, 2000). As such, if the intervention succeeds across both staff and children in fostering the above social-contextual conditions emanating from AGT and SDT, it is expected that staff and consequently children may enhance their intrinsic motivation for learning, persist in their respective activities, and have creativity and performance, as well as increase their self-esteem and general socioemotional health.

To create a mastery-motivational climate at both the preschool and child levels, we will use the instructional strategies put forward by Ames (1992, p.267): (a) provide staff and children with tasks that are meaningful, novel, varied, and that offer reasonable (personal) challenge and focus on self-referenced goals; (b) focus on individual improvement, progress and mastery, value effort, and encourage viewing mistakes as part of learning. AGT has shown to be effective in intervention studies aiming to improve MVPA and motor skills in preschool children (Robinson et al., 2016, 2018; Palmer et al., 2017). Further, grounded in SDT: create an autonomy-, structure-, and relatedness-supportive climate that includes the staff and children in their respective processes of learning and development by allowing for influence in decision-making, giving opportunities to develop responsibility and independence, and structuring their learning and play activities, as well as creating a sense of group belongingness (Ryan and Deci, 2000).

#### The Child Level

The intervention at the child level is derived from theory and evidence relating to the beneficial effects of PA on physical, socioemotional, and cognitive development. Central to the ACTNOW intervention is opportunities for children to engage in a variety of meaningful and joyful *physically active play* activities that support the development of the whole child.

**FIGURE 2:**
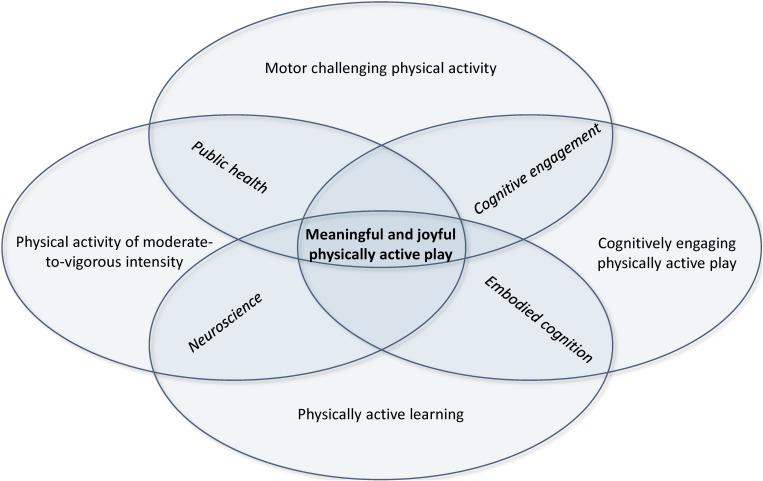
The intervention model at the child level.

The rationale for each of the four core components in the intervention model is based on theory and evidence from different fields of research, including physiological, psychological, and phenomenological perspectives, including both acute and chronic effects of PA, as illustrated in the inner flower in the diagram (Figure 2). Essentially, the left part of the intervention model is focused on the quantitative characteristics of PA, such as the dose (duration, intensity, and/or frequency) of PA. The right part of the model focuses on the qualitative characteristics of the intervention, with emphasis on the type and mode of PA. By the integration of the four core components, we aim to provide children opportunities to increase MVPA across a wide range of motor competencies to enhance development, cognitively engaging play, and physically active learning.

##### Modes of delivery

The intervention will be delivered by the staff through the use of a wide range of physically active play (Table 1); from child-initiated and directed free play at the one end, to adult-initiated child-directed guided play and adult-directed and initiated structured teacher-led PA at the other end (Deena Skolnick et al., 2015). In accordance with our theoretical frameworks for the intervention, guided play gives the child autonomy to decide what to do, what to explore, and how to do it. Hence, the role of the teachers when using guided play is to provide children with an enriched PA environment and scaffold the children’s initiatives and actions (Deena Skolnick et al., 2015; Archer and Siraj, 2017). Such an approach can extend children’s understanding of, for example, spatial terms (e.g., over, behind, next to), shapes, numbers, and increase their movement vocabulary (Deena Skolnick et al., 2015; Archer and Siraj, 2017). Furthermore, preschool teachers will be encouraged to take part in the activities and model ways of moving, as well as scaffolding children in need to overcome physical-motor barriers to ensure that *all* of them develop the necessary competencies to be included in and master the activities (Archer and Siraj, 2017). Reaching every child is a primary goal since PA levels and motor competencies vary considerably among children, and preschools might favor PA in some groups of children more than others (Nilsen et al., 2019b, c).

**TABLE 1:** The four core components of the intervention (child level).

Component	Delivery	Dose
Physical activity of moderate-to-vigorous intensity	The MVPA component will focus on promoting energetic play using gross motor activities involving the whole body, such as running, skipping, and jumping, through: • Short bouts of vigorous PA (e.g., spurts of running). This activity pattern builds on the natural pattern of PA in the early years (Ruiz et al., 2018). • Breaking up outdoor free play to promote structured adult-led MVPA. Because children are most active at the beginning of a play session, a boost with adult-led activity will potentially increase the level of MVPA (Greever et al., 2015).	60 min/day
Motor challenging PA	The motor challenge component will focus on physically active play and movements that challenge children’s motor competencies through: • Various gross motor activities across all motor competence domains; locomotion, object control, and balance. • Manipulation of tasks and environment to meet the children’s optimal challenge point (Guadagnoli and Lee, 2004).	90 min/week
Cognitively engaging physically active play	The cognitively engaging component will focus on physical active play requiring mental engagement through: • Physically active play that taps the executive functions, such as stop tasks, memory tasks, shifting tasks, and creativity tasks (Tomporowski et al., 2015). • Modified group games requiring executive functions, for example, ball games and tag games (Tomporowski et al., 2015).	90 min/week
Physically active learning	The physically active learning component will focus on PA integrated with learning activities through: • Non-task-relevant MVPA that incorporates academic content from the Framework Plan (Norwegian Directorate for Education and Training, 2017). • Task-relevant PA integrated with academic content from the Framework Plan (Norwegian Directorate for Education and Training, 2017).	90 min/week

##### Environment and context

The intervention activities will take place indoors and outdoors, both within and outside of the preschool area (e.g., using a nearby forest, beach, park, playground, etc.). Because more time spent outdoors is associated with increased PA (Bingham et al., 2016; Driediger et al., 2018), and better cognitive and behavioral development (Ulset et al., 2017), preschools will be encouraged to spend ample time outdoors each day. Through the use of various environments, children will have access to play areas with a variety of surfaces (e.g., floor, grass, sand, bark, mud, dry, wet, snow, ice, etc.) and terrain (flat, hilly, rocks, trees, etc.) to support the variability of practice and optimal stimuli of motor and cognitive development (Pesce et al., 2019). The environment, both inside and outside, will be enriched by portable equipment to encourage various movements, including hula-hoops, balls of different sizes and weights, bean bags, sensory slices, cones, and hurdles. Such portable play equipment has been shown to increase MVPA (Driediger et al., 2018; Määttä et al., 2019). Furthermore, equipment such as dice, letters, shapes, and pictures will be provided to incorporate learning goals through physically active play.

Most of the intervention activities will take place in groups of children, facilitating interaction between peers and with staff. Socially oriented physically active play and games are important for the development of socioemotional competence and hence friendships in the early years. Furthermore, physically active play can affect social connectedness and a sense of belonging (Bailey, 2017). Therefore, promotion of such activities is especially important for children showing the least developed social skills (Bailey, 2017).

##### The “physical activity of moderate-to-vigorous intensity” component

The specific amount of PA needed for optimal development and health in the early years is unclear (Timmons et al., 2012; Carson et al., 2017). More intense PA appears to be most favorable to health (Carson et al., 2017), and cognitive and psychosocial development (McNeill et al., 2018). In Norway and internationally, it is recommended that young children spend at least 60 min/day in MVPA (Tetens et al., 2014; World Health Organization [WHO], 2019). There is general concern about low PA levels internationally (Bornstein et al., 2011; Driediger et al., 2018; Truelove et al., 2018), and recent evidence shows that only 55% of 3- to 5-years-old in Norway achieve this amount of MVPA (Nilsen et al., 2019c).

Beyond physical health, a sufficient dose of PA is also hypothesized to be of importance for cognition, with executive function being the most promising outcome (Tomporowski et al., 2008). The general physiological and metabolic mechanisms that are related to aerobic fitness (Best, 2010) are suggested as pathways for how PA affects executive functions (The Aerobic Fitness Hypothesis) (Howie et al., 2015; Howie and Pate, 2018). Research with 9- to 10-years-old children has shown that higher-fit children have better executive function, greater activity in brain regions important for attention and memory, and a positive change in gray matter volume in brain regions implicated in learning (Chaddock et al., 2011). In preschool children, Niederer et al. (2011) found that aerobic fitness predicted attention 9 months later, but such relationships are scarcely investigated in young children.

##### The “Motor challenging physical activity” component

Learning to move is essential for PA, and during the early years, children acquire competence in diverse fundamental movement skills, such as locomotion and object control (Stodden et al., 2008). Motor competence has been suggested to be a primary underlying mechanism that promotes engagement in PA, both acutely and over the long-term. A conceptual model by Stodden et al. (2008) proposes a synergistic relationship among motor competence, perceived motor competence, PA, physical fitness, and weight status. During the early years, the child’s perception of their motor competence is especially important, as children with high perceived motor competence are more persistent and engaged in various activities. Importantly, their perceived motor competence might be weakly correlated to their actual motor competence (Stodden et al., 2008). As such, an intervention aiming to improve motor competence might benefit from building children’s perceptions of their motor competence. Moreover, both AGT and SDT emphasize perceived competence as an important psychological factor (Robinson et al., 2015), which may act as a mediator between actual motor competence and actual/future PA, as well as various developmental outcomes. Robinson et al. (2015) reviewed the proposed links in the conceptual model by Stodden et al. (2008) and concluded that motor competence and PA are weakly to moderately associated in early and middle childhood. This is consistent with results from a large Norwegian study in preschoolers, which extends these results by showing that the association pattern between PA and motor competence is characterized particularly by vigorous intensities (Nilsen et al., 2019a).

Importantly, motor competence will likely not be optimally stimulated through naturally occurring behaviors and thus needs to be taught (Stodden et al., 2008; Robinson et al., 2016). Furthermore, to optimally promote motor competence, it is important to provide challenging activities that keep the children on a positive trajectory of learning and maximize motivation and engagement (Robinson et al., 2015). Consistent with the Challenge Point Framework (Guadagnoli and Lee, 2004), the ACTNOW intervention seeks to adapt intervention activities that are developmentally appropriate and individually adapted in order to meet each child’s optimal challenge point.

Providing children with challenging motor tasks may also be a viable way to affect their executive functions, through increased cognitive engagement (Best, 2010). Motor tasks that are novel or not automatized require the involvement of executive functions (Diamond, 2000; Guadagnoli and Lee, 2004; van der Fels et al., 2015; Pesce et al., 2019), and an interrelation between the brain structures important for motor skills (cerebellum) and the structures important for executive functions (the prefrontal cortex) has been described (Diamond, 2000). The interrelation between these structures is evident by their co-activation during motor and cognitive tasks, their similar developmental timetable, and their common underlying processes such as, for example, planning (Diamond, 2000; Roebers et al., 2014; van der Fels et al., 2015). As such, practicing challenging motor tasks may lead to specific adaptation in brain areas (prefrontal cortex) that support executive functions (Best, 2010). The importance of meeting children’s optimal challenge point to affect their executive functions has been highlighted in the literature (Pesce et al., 2013, 2019; Tomporowski et al., 2015; Diamond and Ling, 2016). Some evidence also exists for added value of motor skill tasks to executive functions beyond the effects of MVPA (Chang et al., 2013; Koutsandreou et al., 2016).

##### The “Cognitively engaging physically active play” component

Increasing the complexity of the PA context through structured games or active play, which engage core executive functions, is a viable way to increase the cognitive demands of PA and hence promote executive functions (Best, 2010; Pesce, 2012; Tomporowski et al., 2015). It has been suggested that engaging executive functions through play can confer benefits to other domains where executive functions are needed (Best, 2010). In group games, the learning environment is in constant change, and the child needs to adapt his/her movement in relation to these dynamic changes. In doing so, the child uses his/her executive functions.

Furthermore, manipulation of activities can increase demands on executive functions. Such manipulation is important, as executive functions need to be continuously challenged in order to be improved (Tomporowski et al., 2015; Diamond and Ling, 2016). In regard to the optimal Challenge Point Framework (Guadagnoli and Lee, 2004), the cognitive load of an activity needs to be tailored to each child. Previous research has shown that children with different abilities may benefit from different activities. For example, Pesce et al. (2013) showed that a cognitively enriched PA program facilitated development of attention in typically developing children (aged 5–10) but not in children with Developmental Coordination Disorder.

##### The “Physically active learning” component

The physically active learning component will integrate PA with different learning areas in *The Norwegian Framework Plan for Kindergartens* (Norwegian Directorate for Education and Training, 2017). Learning areas in the Framework plan are: (*1) communication, language, and text; (2) body, movement, food, and health; (3) art, culture, and creativity; (4) nature, environment, and technology; (5) quantity, spaces, and shapes; (6) ethics, religion, and philosophy; and (7) local community and society* (Norwegian Directorate for Education and Training, 2017). The physically active learning component derives from the research fields of both acute exercise and embodied cognition. From the exercise and cognition field, the physiological effects of acute PA might facilitate the learning process of academic content by increased arousal. For example, acute PA of a sufficient intensity/dose can raise the concentration of neurotransmitters and cortisol to levels that have positive effects on cognition (McMorris and Hale, 2012; Koutsandréou et al., 2016). Bouts of PA with academic content have shown improved on-task behavior in school settings (Mahar et al., 2006; Grieco et al., 2016). As such, gross motor MVPA coupled with academic content will be promoted in the intervention.

From the perspective of embodied cognition, the relevance of PA for the academic content is of importance. Gesturing or PA that are tightly coupled to the academic content might off-load demands on working memory during learning, through experiencing complementary content through several senses that use different memory traces. As such, it is hypothesized that the learning trace will be of higher quality, and as such better learned (Mavilidi et al., 2018b). Some of the intervention activities will be tightly coupled to the learning content, for example, by forming letters, shapes, and figures with the children’s bodies. Several studies in preschool children have successfully used task-relevant PA to learn academic content in, for example, language/literacy (Kirk et al., 2014; Mavilidi et al., 2015), numeracy (Schmidt et al., 2015), and science (Mavilidi et al., 2017).

#### The Preschool Level

The staff professional development is described in Table 2. As described above, a flexible intervention that includes co-creation and continual improvement with staff is recommended to facilitate sustainable change in educational contexts (Routen et al., 2017). The ACTNOW intervention will therefore be adjusted to the needs and resources in each preschool. Alongside the involvement of directors and teachers in the professional development, the research group will work in partnership with each preschool and its entire staff to ensure support and commitment, not only among those enrolled in the professional development. As recommended by the literature on health promotion assessment and interweaving (Springer et al., 2017), the process among staff will begin by involving all in an analysis of the preschool’s existing PA policies and practices as well as an assessment of staff and children’s needs and resources related to PA, and thus their readiness for change. Based on the assessment, staff will develop a written PA policy with set priorities and objectives, which will incorporate both intervention levels, including the four core components for children. Depending on each preschool’s current practice, the elements of the intervention will add to, extend, and/or integrate with and improve existing activities in line with the Theory of Expanded, Extended, and Enhanced Opportunities (Beets et al., 2016), which corresponds with concepts of organizational learning (Nonaka, 1994; Wells, 1999; Moilanen, 2005). In collaboration with the researchers, the next phase will involve developing strategies for the implementation and integration of the new policy and practices into the preschool’s daily practice (Fitzgerald and Theilheimer, 2013; Heikka et al., 2019). The final phase will consist of monitoring and evaluation, revision, and learning. Given that the principal/director in educational contexts plays a pivotal role in the development and adoption of interventions (Roberts et al., 2015), the preschool director, together with the teachers taking part in the professional development, will be in charge of leading the rest of the staff through the process (Boe and Hognestad, 2017). The researchers will support the implementation process through email, telephone, and visits to each preschool during the intervention period. In addition, a customized website with teaching resources will be provided.

**TABLE 2:** Overview of the professional development (preschool level).

Structure	The professional development will be structured as a 15-credit continuing education module at a master’s degree level delivered over 7 months. However, the staff can choose whether they will complete the exam and achieve credits or complete the professional development without credits. Seminars and webinars total 6 days face-to-face (3 days on campus and 3 days locally in each preschool) and multiple webinars, amounting to approximately 50 h in total.

Requirements for participation	We will request the preschool director and minimum one teacher from each classroom to participate. Skilled workers/assistants and others will primarily be involved locally through the processes initiated and facilitated by the director and teacher(s). Yet, preschools can choose to let skilled workers/assistants participate in the professional development, depending on their needs and resources.

Aims/content	The aims of the professional development are to increase the preschool directors’ and teachers’ competences regarding 1. the importance of physically active play and its relevance for child development, and how to integrate more physically active play (the core components) into the preschool’s practice; and 2. planning and implementation of interventions/changing practice, and thus facilitate the ACTNOW implementation process within the entire preschool and its staff.

Process	The professional development has three phases **Phase 1: Setting the stage (2 months)** The professional development will start by an intensive 2-day face-to-face seminar. One part of the seminar will focus on the intervention at the child level, with both practical and theoretical sessions regarding the four core components, as well as general topics on PA and physically active play, motor competence, cognitive development, and didactics. The other part of the seminar will focus on the process of the development work among the preschool staff. The seminar will be followed by a 1-day visit to each preschool by a member of the research group. This visit will include a half-day observation of practice and a 2 h staff meeting, planned by the preschools, including discussion of each preschool’s specific PA practice, contextual factors, needs, resources, etc. During this first phase, the directors/teachers will be responsible for drafting a model on how to integrate the four core components into their daily practice, which will be subject to feedback and discussion in an individual webinar with the research group.
	**Phase 2: Customize the intervention for sustainability (3 months)** The second phase of the professional development will focus on the implementation of each preschool’s intervention model at the child level. Based on the initial experiences and discussions in Phase 1, each preschool model will be revised, if necessary, and further developed to meet with the preschool practice and the prescribed intervention dose. During this second phase, multiple webinars will be given to extend the lectures and discussions in Phase 1. A group discussion will be arranged, where participants and the research group meet to share the preschools’ experiences regarding the implementation process.
	**Phase 3: Reflect on and evaluate the intervention (2 months)** The third phase of the professional development will focus on reflection and evaluation of the changed preschool practice regarding the implementation of the preschool’s intervention model. Multiple webinars will be given, in addition to a 1-day visit in each preschool, with a new half-day observation, followed by a joint reflection in the whole staff group about the developmental work and (hopefully) changed practices. The professional development will end with a final full-day session of experience sharing among all intervention preschools, focusing on lessons learned and the way forward.

### Protocol and Randomization

**FIGURE 3:**
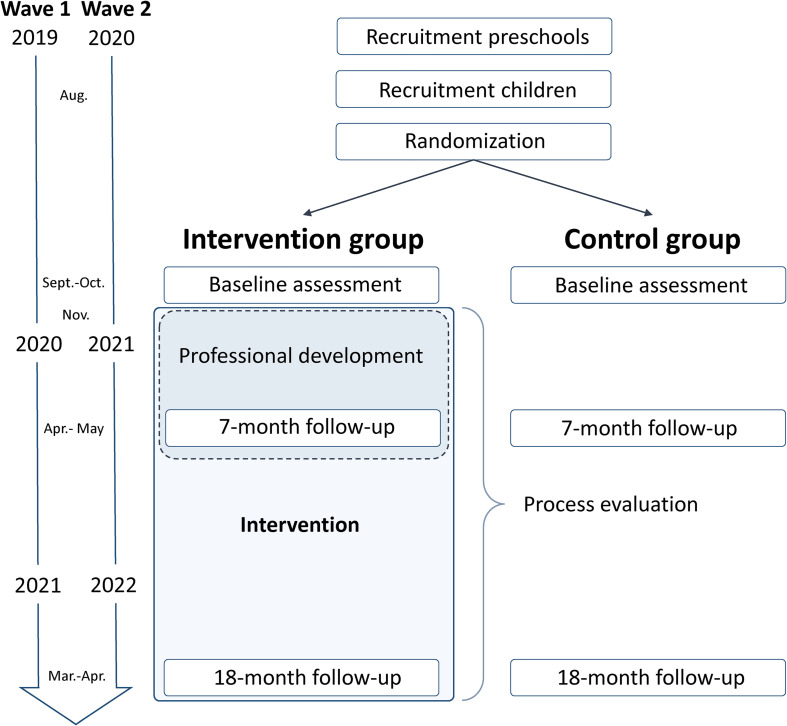
Flow chart of the study.

ACTNOW will have two waves involving different cohorts, the first running from August 2019 to June 2021, and the second from August 2020 to June 2022 (Figure 3). The professional development provided in Wave 2 will be adjusted based on experiences and findings from Wave 1. Data collection will have three main stages; pre-testing performed before randomization, 7-month follow-up performed at the end of the professional development, and 18-month follow-up performed 1 year after staff have completed the professional development. In addition, process evaluation measures will be taken throughout the study.

We will cluster randomize at the preschool level, since we regard delivery of the intervention on the preschool level the only practical approach. A third party (Morten Wang Fagerland, Norwegian School of Sport Sciences) will randomize ≈ 60 preschools to the intervention group (≈ 30 preschools) or the standard of care control group (≈ 30 preschools). Randomization will be done separately in the two study waves after completing recruitment and before initiating baseline data collection using a random number generator (Stata/SE 15.1, StataCorp LLC, College Station, TX, United States). We will use block randomization (randomly chosen block sizes of 4 and 6) and a 1:1 ratio for the two intervention arms. Blinding of participants and preschools is not possible due to the nature of the experiment. To limit potential biases, the fewest possible assessors involved in project management, data collection, and statistical analysis will have knowledge about the group assignment and the study hypotheses.

### Participants

We will invite all preschools (both public and private) in multiple regions in the county of Sogn og Fjordane, Norway, having ≥ six 3- to 4-year-old children (i.e., requiring a minimum number of children available for 2-year follow-up) to participate in the study. We will invite all 3- to 5-years-old children enrolled in these preschools (Wave 1: children born 2014–2016; Wave 2: children born 2015–2017). Children who are unable to take part in normal everyday PA as promoted by the preschools as part of the intervention will be excluded from the analyses.

Sample-size calculations are derived from standardized ESs in meta-analyses of interventions investigating preschoolers’ objectively measured PAs (Finch et al., 2016) and motor competences (Wick et al., 2017), and schoolchildren’s cognition (Alvarez-Bueno et al., 2017b) and learning (Alvarez-Bueno et al., 2017a). Preschool interventions have shown small ESs for long-term interventions delivered by preschool staff targeting PA [ES = 0.07 (three studies, > 6 months) and 0.27 (10 studies, delivered by teachers)] (Finch et al., 2016) and motor competence [ES = 0.32 (six studies, vs 6 months) and 0.41 (eight studies, delivered by teachers)] (Wick et al., 2017). Rather similar ESs are shown for cognition (0.11-0.30) (Alvarez-Bueno et al., 2017b) and learning outcomes (0.16-0.26) (Alvarez-Bueno et al., 2017a) in older children (4-18 years). Derived from a conservative sample-size calculation using standard formulas, including correction for the cluster RCT design, we aim to recruit a minimum of 60 preschools and 800 3- to 4-year-old children to the study. This sample size will allow for uncovering statistically significant standardized ESs (Cohen’s d) of 0.25-0.30 at the 18-month follow-up, given *ɑ* = 0.05, 1-β = 80%, group ratio 1:1, correlation of repeated measurements = 0.6-0.7, intra-class correlation (ICC) across clusters = 0.05-0.10, children per cluster = 13 accounting for 30% attrition among children (median number of 3- to 4-years-old in preschools in Sogn og Fjordane ≈ 18), and coefficient of variation for cluster sample size = 0.65 (Eldridge et al., 2006). Thus, we will require ≈ 400 children and 30 preschools for analysis in each group (intervention; control), given a design effect correction factor = 1.85-2.69. The ICC is estimated using a mean ICC = 0.05 for trials investigating objectively measured PA levels in preschoolers (Finch et al., 2016), results from the Sogn og Fjordane Preschool Physical Activity Study: ICC ≤ 0.10 for PA (Nilsen et al., 2019b) and ICC ≤ 0.05 for motor competence (unpublished), and ICC = 0.07-0.09 for academic performance in schoolchildren (Resaland et al., 2016). Because the sample-size calculation is performed for the 18-month follow-up of 3- to 4-years-old, we will have increased power at the 7-month follow-up, as we also include 5-years-old (minimum total *n* ≈ 1,200 children); ESs as low as 0.20 can be detected at the 7-month follow-up under the assumptions specified above.

### Data and Measurements

#### The Child Level

##### Physical activity

We will assess PA objectively over 7 consecutive days using the ActiGraph GT3X + accelerometer (John and Freedson, 2012), which is the most used and validated accelerometer in the field (De Vries et al., 2009; O’Brien et al., 2018). Children will be instructed to wear the accelerometer on the right hip 24 h per day, except during water activities (swimming, showering). Units will be initialized at a sampling rate of 30 Hz, and files will be analyzed at 1-s epochs using the KineSoft analytical software version 3.3.80 (KineSoft, Loughborough, United Kingdom) to correctly capture the lower and higher intensity levels (Aadland et al., 2018a, 2019). In all analyses, consecutive periods of ≥ 20 min of zero counts will be defined as non-wear time (Esliger et al., 2005; Cain et al., 2013). Results will be reported for the overall PA level [counts per minute (cpm)], as well as minutes per day spent sedentary (<100 cpm), in light PA (100–2295 cpm), in moderate PA (2296–4011 cpm), in vigorous PA (≥4012 cpm), and in MVPA (≥ 2296 cpm), determined using the previously established and validated Evenson et al. cut points (Evenson et al., 2008; Trost et al., 2011). As appropriate, sensitivity analyses will be conducted using the Pate et al. cut points (Pate et al., 2006).

In addition to the “standard” PA intensity profile, we will create a dataset having a higher resolution using 33 PA variables of total time (min/day) to capture movement in narrow intensity intervals throughout the spectrum; 0–99, 100–249, 250–499, 500–999, 1,000–1,499, …, 14,500–14,999, and ≥ 15,000 cpm. As shown previously (Aadland et al., 2018b; Nilsen et al., 2019a), this dataset will be used to investigate the PA signature associated with relevant outcomes.

PA during the whole day, week, and weekend days, as well as PA during care hours (08:30–15:30) and afternoon hours (15:30–23:59) on weekdays only, will be analyzed to specifically assess intervention effects during care hours and to assess possible compensatory behavior outside the preschool domain (Gomersall et al., 2013; Nilsen et al., 2019b). Data for a full day will be analyzed with wear-time requirements of ≥ 8 h/day and ≥ 3 weekdays + ≥ 1 weekend day, whereas ≥ 5 and ≥ 3 h/day of monitoring for ≥ 3 weekdays will be used as wear requirements for care hours and afternoons, respectively.

Screen time will be assessed using a parent questionnaire adapted from the Sunrise study protocol (Sunrise, 2020). Measurement properties of the questionnaire will be documented.

##### Physical fitness and motor skills

The fitness assessment in the ACTNOW study is based on three items (handgrip strength, standing long jump, and speed-agility) from the Assessing FITness in PREschoolers (PREFIT) battery (Ortega et al., 2015). PREFIT is an adaptation of the ALPHA-Fitness: field-based fitness tests for the assessment of health-related physical fitness in children and adolescents, and have demonstrated good reliability in young children (Castro-Pinero et al., 2010; Artero et al., 2011; Ruiz et al., 2011). Hand-grip strength will be measured with a hand dynamometer with adjustable grip (TKK 5001 Grip A, analog model (Cadenas-Sanchez et al., 2016), measurement range 0–100; Takey, Tokio Japan), and a grip−span of 4.0 cm (Sanchez-Delgado et al., 2015). Lower body explosive strength will be measured using standing long jump, where children are instructed to jump as far as possible, measured in centimeters. Speed-agility will be assessed using a shuttle running test where children are instructed to run 4 × 10 m as fast as possible, measured in seconds.

To evaluate fundamental motor skills (FMS), we developed a test battery guided by the “Test of Gross Motor Development 3” (TGMD-3) (Ulrich, 2013, 2019). TGMD-3 is designed for children aged 3–10 years, and originally based on observation of children’s movements across 13 tasks within the two domains: locomotion (run, skip, slide, gallop, hop, and horizontal jump) and ball/object control (hereafter referred to as “object control”) (overhand throw, underhand throw, catch, dribble, kick, one-hand strike, and two-hand strike). We modified this test battery to reduce the participant and researcher burden, and at the same time cover the three main domains of FMS by including balance skills (Sun et al., 2010). We therefore included six movement items from the TGMD-3 battery (run, horizontal jump, hop, catch, overhand throw, and kick), in addition to three movement items within the balance domain (single leg standing, walking line forward, and walking line backward) from the Preschooler Gross Motor Quality Scale (PGMQ) proposed by Sun et al. (2010). The specific items were selected based on their relevance (e.g., some of the movement items in the TGMD-3, like the baseball strike and dribble, are less common and therefore less relevant in assessments of Norwegian children), and variety (e.g., including object control skills related to both hands and feet, and adding both static and dynamic balance tests) to broadly capture children’s skills within the three FMS domains. We have previously demonstrated good inter-rater reliability for this FMS battery (Nilsen et al., 2019a).

Children’s fitness and FMS will be evaluated in small groups (4–5 children) during preschool hours, in a safe environment with enough space to move freely. Each child performed each task twice, and skills will be completed in a standardized order, using approximately 40 min per group. The test teams consist of one instructor who provide a verbal description and demonstration of the required skill, while a separate assessor observe and score the performance. The physical fitness and FMS tests are administered and scored according to the TGMD-3 (locomotor and object control skills), PGMQ (balance skills), and PREFIT-protocols (hand grip strength, standing long jump, speed-agility). Briefly, each of the nine FMS movement tasks is described by three to four performance criteria, which is scored 0 or 1 if a criterion is absent or present, respectively. Scores are summed for each task and each domain.

##### Anthropometry and demography

We will assess children’s body mass, height, and waist circumference according to the PREFIT battery (Ortega et al., 2015). Body weight will be measured to the nearest 0.1 kg using an electronic scale (Seca 899, SECA GmbH, Hamburg, Germany), and height will be measured to the nearest 0.1 cm with a portable stadiometer (Seca 217, SECA GmbH, Hamburg, Germany). Body mass index (kg/m^2^) is calculated and children classified as normal weight, overweight, or obese based on criteria proposed by Cole et al. (2000). Waist circumference will be measured twice to the nearest 0.1 cm at the level of the umbilical zone. Demography includes the variables socioeconomic status (parents’ education and income), children’s and parents’ origins, children’s birth weights, parents’ weight status, and parents’ PAs, and they will be measured by parent questionnaires.

##### Sleep

Children’s sleep will be assessed using 24 h accelerometry and a parent questionnaire. Accelerometry has been applied to assess sleep in multiple studies in preschoolers and older children (Galland et al., 2018), although procedures for assessment of sleep are less developed than for movement behaviors (Migueles et al., 2017). Most of these studies have applied wrist-worn accelerometers (Migueles et al., 2017; Galland et al., 2018), but there are ongoing efforts to assess and develop measurement properties of sleep for waist-worn accelerometry (Hjorth et al., 2012; Zinkhan et al., 2014; Barreira et al., 2015). There is currently no validated accelerometry-based sleep scoring algorithm for preschoolers (Migueles et al., 2017). We will therefore use the scoring algorithm by Sadeh et al. (1994), which is developed for assessment of sleep in children and youth, and incorporated by ActiGraph (ActiLife), as the starting point. However, since this algorithm was developed for wrist-worn accelerometers, using it with waist-worn accelerometers should be done with care since results are not directly comparable (Hjorth et al., 2012; Zinkhan et al., 2014). Alternative scoring protocols developed with or validated for waist-worn accelerometers, for example as suggested by Barreira et al. (2015), will therefore be used as appropriate, if improved measurement properties can be demonstrated in preschoolers.

In the questionnaire, adapted from the Sunrise study protocol (Sunrise, 2020), parents are asked to report children’s sleep duration (including nap time), when they go to bed, when they wake up, and whether these patterns are consistent. Furthermore, they will rate children’s sleep quality (1–7 scale). Measurement properties of the questionnaire will be documented.

##### Socioemotional health, self-regulation, executive function, and learning

We will adapt several validated tests to examine different aspects of socioemotional health, self-regulation, executive functions, and learning. Yet, there exists little agreement on the optimal measures and their configuration for capturing important aspects of early cognitive development (Garon et al., 2008; Howard and Melhuish, 2017).

To assess **socioemotional health,** we will ask the teachers to fill out the Strengths and Difficulties Questionnaire (SDQ) for each child. The SDQ is a brief measure of psychosocial strengths and problems in 3- to 16-years-old children. The SDQ asks about 25 attributes (10 positive, 14 negative, and 1 neutral) divided between five subscales of five items each: (*1) Emotional Symptoms Scale; (2) Conduct Problems Scale; (3) Hyperactivity Scale; (4) Peer Problems Scale;* and (*5) Prosocial Scale*. All but the last scale are also summed to give a total difficulties score. SDQ uses a 3-point Likert scale to indicate the extent to which each attribute applies to a child (“not true,” “somewhat true,” or “certainly true”) (Goodman, 1997, 2001). The construct validity of the SDQ has been demonstrated in several studies (Goodman, 2001; Stone et al., 2010; Mieloo et al., 2012), and the reliability and validity of the SDQ has been shown to be satisfactory (Goodman, 2001; Stone et al., 2010). The teacher version of the SDQ has shown both higher internal consistency and test-retest reliability compared to the parent version for preschool and schoolchildren (Stone et al., 2010; Mieloo et al., 2012).

**Self-regulation** will be assessed both by teacher-report and a structured observation (direct measure) of a child’s performance. Teachers will be asked to fill out the 33-item Child Self-regulation and Behavior Questionnaire form from the Early Years Toolbox (EYT) (Howard and Melhuish, 2017). The Child Self-regulation and Behavior Questionnaire generates subscales on cognitive, behavioral, and emotional self-regulation, in addition to sociability, prosocial behavior, externalizing and internalizing problems, which overlaps with the SDQ. Each item asks the teacher to evaluate the frequency of target behaviors of each child on a scale from 1 (not true) to 5 (certainly true). The subscales of the questionnaire have been shown to be reliable in preschoolers (Howard and Melhuish, 2017).

The directly assessed Head-Toes-Knees-Shoulders task assesses the ability of a child to use and integrate the executive functions to control and direct actions, pay attention, and remember instructions (Cameron Ponitz et al., 2008; Ponitz et al., 2009; Cameron et al., 2012; von Suchodoletz et al., 2013). To respond correctly, the task requires that children listen to instructions, remember and execute gross motor movements in relation to directions, and inhibit pre-potent incorrect responses. In the first part (10 items), two rules are paired: “touch your head” with “touch your toes.” In the second part (10 items), two more paired rules are added: “touch your knees” with “touch your shoulders” (Ponitz et al., 2009). Children are instructed to perform the opposite of the dominant response. Hence, the command to “touch your head” requires the children to touch their toes, and the command to “touch your shoulders” requires the children to touch their knees. If the children reach a minimum performance threshold of 4 points on both of these levels (2 points are given for a correct response, and 1 point for a self-corrected response), a third section (10 items) will be given, where the paired rules are switched (i.e., head goes with knees and shoulders go with toes) (McClelland et al., 2014). Instructions will be given verbally in a fixed order, without feedback. Preceding each level, four practice trials and up to three attempts on each are provided to familiarize with the test (Cameron Ponitz et al., 2008). The Head-Toes-Knees-Shoulders task is quick to administer, requires few materials, and has shown construct validity in European samples (von Suchodoletz et al., 2013).

We will assess all three key **executive functions** identified by Miyake et al. (2000): inhibition, working memory, and cognitive flexibility. These three executive functions will be measured using assessments from the iPad-based EYT (Howard and Melhuish, 2017). EYT has shown good reliability, convergent validity with existing measures, and developmental sensitivity (Howard and Melhuish, 2017). The EYT is developed for preschool children (which contrasts the majority of measures that are modified adult versions), with emphasis on being (a) developmentally appropriate, (b) developmentally sensitive, (c) brief, (d) engaging, (e) technologically dynamic without introducing effects of technological expertise, and (f) internationally applicable. The EYT is available on iTunes App Store. We embedded a Norwegian version to ensure its application in the children’s native language.

To measure the visual-spatial working memory we will use *“The Mr. Ant task”* (Howard and Melhuish, 2017). In this task, children are asked to remember spatial locations of “sticker/s” that are placed on Mr. Ant’s body. The children then, after a brief retention interval, tap the spatial locations where they believe the stickers were on Mr. Ant. The test trials increase in difficulty by increasing the number of stickers on Mr. Ant’s body (progressing from one to eight stickers). All trials progress as follows: (a) Mr. Ant presents with *n* colored stickers for 5 s, (b) the screen is blank for 4 s, and then (c) an image of Mr. Ant without stickers – along with an auditory prompt to recall where the stickers were – is presented until the child’s response is complete. The task continues until completion, or until failure on all three trials at the same level of difficulty. Children are given instruction and three practice trials to familiarize them with task requirements prior to administration of test trials. Working memory will be indexed by a point score calculated as follows: starting from Level 1, 1 point for each passed level (at least two of the three trials performed accurately), plus 1/3 of a point for all correct trials thereafter (Howard and Melhuish, 2017).

To measure inhibition, we will use *“The Go/No-Go task”* (Howard and Melhuish, 2017). In this task, the children are asked to catch the fish (Go trial) by tapping the screen when they see a fish, and to avoid the shark (No-Go trial) by not tapping the screen when they see a shark. Because the majority of the stimuli are Go trials (80% fish), this generates a pre-potent response to tap the screen, requiring the children to inhibit this response (not tap) in No-Go trials (20% sharks). The children are given instructions and practice to familiarize with requirements as follows: go instructions, followed by five practice Go-trials; No-Go instructions followed by five practice No-Go trials; combined Go/No-Go instructions followed by a mixed block of 10 practice trials (80% Go-trials); and a recap of instructions. Feedback in the form of auditory tones is provided on all practice trials. The task proceeds with 75 stimuli divided evenly in three 1-min test blocks (each separated by a short break and a reiteration of instructions). Blocks always begin with a Go stimulus, and no more than two successive trials are No-Go stimuli. Each trial involves a presentation of an animated stimulus (i.e., fish or shark) for 1,500 ms, separated by a 1,000 ms interstimulus interval. The outcome is an impulse control score that is the product of proportional Go and No-Go accuracy (Howard and Melhuish, 2017).

Cognitive flexibility/shifting will be measured by the “*The Card Sorting task.”* This task asks the children to sort red rabbits and blue boats by either color or shape, into two locations (identified by a blue rabbit and a red boat). As the card sorting rules alternate, the children must switch between rules. The children are first given a demonstration trial and two practice trials. Then they are asked to sort stimuli by one dimension for six trials (with the same stimulus never presented more than twice in a row). In the subsequent post-switch phase, children are required to sort by the other sorting dimension, as prompted by auditory instructions preceding post-switch test trials. If the child correctly sorts at least five of the six pre- and post-switch stimuli, they proceed to a border phase of the task. In this phase, children are required to sort by color if the card has a black border or sort by shape if the card has no black border, preceded by a demonstration trial and two practice trials. This sorting rule is reiterated prior to all test trials. Scoring represents the number of correct sorts after the pre-switch phase (Howard and Melhuish, 2017).

As a proxy for **learning** we will use “*The Expressive Vocabulary”* for language development (Howard and Melhuish, 2017) and *“The Numbers task”* for early math skills (manuscript not published), both from the EYT. Both measures have shown good convergent validity and reliability (Howard and Melhuish, 2017). In the Expressive Vocabulary task, consisting of 54 items, children are asked to verbally produce a correct label for the depicted nouns and verbs. The administrator records the response directly into the iPad app (correct/not correct). In cases of an incorrect label, the administrator will prompt the children by asking, “What else might this be called?” until either a correct production or an indication that the child is unable to produce the target word. A six-item failure stop rule will be used to minimize administration time. Expressive Vocabulary performance will be scored as overall accuracy (Howard and Melhuish, 2017).

The Numbers task consists of 79 items pertaining to numerical concepts (e.g., many), spatial and measurement concepts (e.g., tallest), counting subset (e.g., counting dogs interspersed with cats), matching digits and quantities (e.g., understanding the correspondence between: spoken number *two*, the digit *2*, and its corresponding quantity), number order (e.g., identifying the missing number in a number line), ordinality (e.g., identifying higher/lower numbers), cardinality (e.g., identifying what is 1st, 2nd, etc.), subitizing (e.g., a rapid appraisal of quantities as having more or less), patterning (e.g., identifying and completing a pattern), and numerical word problems and equations. In this task, children respond to each item either by tapping the iPad screen or verbally answer auditory questions in the app (which is recorded directly in the app by the administrator). The app integrates automated start rules based on the age of the child, and stop rules after five consecutive incorrect responses.

#### The Preschool Level

The aim of the process evaluation is to explore how staff in the intervention preschools respond to their enrollment in professional development and how the proposed new way of working with PA gains traction in different contexts. We aim to explore the processes and mechanisms through which professional development, alongside working in partnership with researchers, influence the way staff organize and integrate PA practices into their daily operations. We will also explore *if* and *why* intervention effects may vary between preschools. The flexible intervention approach, and thus the variation in activities (likely) taking place in each preschool, means that an examination of typically quantitative measures of implementation such as fidelity, dosage, and adherence might be less feasible and informative. Hence, the process evaluation includes complementary qualitative and quantitative measures of implementation to support the evaluation of the intervention’s ability to change practice, as also recommended previously (Bonell et al., 2012; Pesce et al., 2016).

##### Staff experiences and perspectives

To provide in-depth accounts of how the intervention preschools develop and implement PA into their daily operations, all intervention preschools will be invited to participate in a qualitative study. Data will be collected using semi-structured in-depth interviews and focus groups. More specifically, in-depth interviews will be used to explore the views of the teachers enrolled in the professional development regarding the relevance and delivery of it. For example, teachers will be asked about how they make sense and engage with the central components of the ACTNOW professional development program. Further, in-depth interviews will be used to explore the directors’ and teachers’ roles in leading the development work taking place in each preschool. For example, questions will be asked to explore how they perceive their role in driving the intervention and the preschool’s PA practices forward as well as what actions and procedures they plan to undertake to lead the process. Focus groups will be used to explore how *all* staff perceive the role of environmental mechanisms in shaping the integration of PA practices into their daily operations. More specifically, staff will be asked about their collective understanding of the intervention and its core components. They will also be asked about their willingness to engage in the developmental work taking place in each preschool and what mechanisms that either promote or hinder engagement and participation.

The literature on implementation, in particular the Normalization Process Theory (May and Finch, 2009), and the characteristics of the ACTNOW project and logic model, have been used to inform the interview guides. The interview guides will be revised and adjusted during data collection to allow for the inclusion of case-specific questions and more detailed exploration of emerging themes. As recommended by Durlak (2016), data will be collected at several time points to allow for an examination of the process of implementation over time. Therefore, the intervention preschools will be approached twice while staff participate in the professional development during Year 1 (once at the beginning and once at the end of the period) and once after its completion during Year 2.

Informed by the findings from the qualitative study conducted during the first wave, we will also develop a survey that will be administered during Wave 2 to all members of staff in the intervention preschools. The aim of this survey is to develop theories about how the identified mechanisms in the qualitative findings play out in contexts with different preschools, staff, and children.

##### Evaluation of written plans

As part of the professional development, we will work with and strongly advise all preschools to anchor the child-level core components of the intervention in their written policies and plans. This work will extend the assessment of existing policies and practices as well as the customized intervention aims and models developed in Phase 1 of the education module. We will use these plans to assess to what extent the intervention is integrated in the preschools’ policies and daily operation over the short- and long-term.

##### Participation and engagement in professional development

Participation in the professional development courses and lectures, as well as completion of tasks related to the implementation process, for example, making aims, drafting models of how the preschool will work to incorporate the intervention, and experience sharing, will be logged to allow for a thorough evaluation of the intervention implementation.

##### Observation of the movement environment

The preschools’ movement environment quality and educational practice will be assessed using the Movement Environment Rating Scale (MOVERS) (Archer and Siraj, 2017) observational scale. This scale captures the quality of the early learning environment and practice based on the presence or absence of high-quality experiences and practices identified as being influential for children’s later outcomes. The scale is completed by highly trained and reliable raters through a fly-on-the-wall observation of a preschool room, conducted across a full day and is supplemented by a review of documentation and discussion with an educational leader. All rooms will be scored at baseline, whereas the scale will be used to support and evaluate the implementation process in the intervention group at later time points.

The MOVERS consists of four subscales relating to quality of experiences and practices related to children’s physical development, namely: (*1) curriculum, environment and resources for physical development* (4 items); (*2) pedagogy for physical development* (3 items); (*3) supporting physical activity and critical thinking* (3 items); and (*4) parents/carers and staff* (1 item). The score ranges from 1 to 7, where 1 = inadequate, 3 = minimal, 5 = good, and 7 = excellent. These quality rating scores are determined by in-balance judgments of the pattern of presence or absence of quality indicators for each item. The MOVERS has been used and validated outside Norway, and we will consider whether it needs adapting to the Norwegian context during the project period.

##### Staff Physical activity

Staff PA will be assessed using accelerometry, which will be performed simultaneously with the three child measurements. We will use the same procedures as detailed for children above, except using another criterion for non-wear time and other cut points to determine intensity-specific PA. Consecutive periods of ≥ 60 min of zero counts, allowing for 1–2 min of non-zero counts, will be defined as non-wear time, according to previous studies in adults (Troiano et al., 2008; Hansen et al., 2012). Results will be reported for overall PA level (cpm), as well as minutes per day spent sedentary (<100 cpm), in light PA (100–2,019 cpm), in moderate PA (2,020–5,998 cpm), in vigorous PA (≥ 5,999 cpm), and in MVPA (≥ 2,020 cpm), determined using the previously established Troiano cut points (Metzger et al., 2008). Consistent with children’s activity patterns and handling of child data, results will primarily be analyzed using short epochs (1 s) to allow for capturing short intermittent bursts of PA. Sensitivity analyses using 60-s epochs will be performed as needed for comparison with previous adults studies (Troiano et al., 2008; Hansen et al., 2012).

#### Data Management

High-quality data collection, data management, and data analysis is of crucial importance to ensure valid study results and secure privacy protection of participants. Prior to the data collection, all assessors will perform thorough training in how to instruct and score children on the different measures to ensure high interrater reliability and minimal measurement error.

Non-digital data on main child-level outcomes (anthropometry, physical fitness, motor competence, and self-regulation) will be double-checked for 100% of cases and triple-checked for a random sample of 10% of cases. Other sources of non-digital data will be double-checked using a random sample of 25% of preschools. After the thorough quality control of data, the database will be locked prior to opening the group allocation (1- and 2-years follow-up) and prior to performing any statistical analysis.

Data handling and storage are confidential and will be managed according to the Western Norway University of Applied Sciences internal control system in accordance with Norwegian privacy protection regulations/the General Data Protection Regulation (EU 2016/679).

### Analysis Plan

#### The Child Level

##### Primary aim

The main analyses of child-level effects will employ an intention-to-treat principle, thus including all subjects and preschools originally allocated to the respective groups, to test the study’s effectiveness (Polit and Gillespie, 2010). However, as shown for the school setting (Donnelly et al., 2009), there are strong arguments for including per-protocol analyses for the examination of efficacy. Therefore, we will perform secondary analyses limited to preschools that exhibit acceptable intervention adherence. Missing data will be sought, minimized, detailed, and handled in line with suggested guidelines (Little et al., 2012). Attrition analyses will be performed to investigate whether missing data are related to child characteristics. Missing data will be handled by the use of linear mixed models (LMMs) and structural equation modeling (SEM) (Enders, 2011; Little, 2013).

Intervention effects will be evaluated by testing for time^∗^group interactions for all outcomes. The per-protocol analysis will be adjusted for differences between the groups, as appropriate. LMM and multilevel SEM for mediation analyses (SEM) (Little, 2013) will be used to account for clustering of observations on the preschool level in all analyses. A two-sided *p* ≤ 0.05 is considered statistically significant; however, both ESs and patterns of effects across variables will be considered of greater importance than *p*-values when drawing study conclusions. Consistent with this approach, *p*-values will not be adjusted for multiple testing. Main analyses will be performed using IBM SPSS v. 25 (IBM SPSS Statistics for Windows, Armonk, NY: IBM Corp., United States) or later versions and MPLUS v. 8 or later versions (Muthén and Muthén, Los Angeles, United States). Reporting will be done in accordance with the CONSORT statement (Campbell et al., 2012).

We regard children’s cognition as the main study outcome. However, we include several aspects of cognition in the present study (socioemotional health, self-regulation, executive functions, and learning), of which designating one focused outcome *a priori* is challenging and inappropriate based on the current level of evidence of effects of PA in young children (Carson et al., 2016; Alvarez-Bueno et al., 2017a, b; Singh et al., 2019). These constructs will be analyzed as separate variables, to retain their unique information, as well as latent variables, to remove measurement error and establish broader constructs, and thus obtain both specific as well as broader knowledge about the effect of PA on cognitive outcomes. Children’s PA, physical fitness, motor competence, adiposity, and staff PA are regarded secondary outcomes.

##### Secondary aim

###### Secondary analyses of intervention effects

*A priori*-defined moderation and mediation analyses will be performed to study child characteristics and pathways that may be important for the effect of the intervention. For example, evidence shows that PA interventions in school are most beneficial for those most in need (Resaland et al., 2016, 2018a) and possibly more beneficial for boys than for girls (Resaland et al., 2018b). Thus, we will test for effect moderation by sex, age, socioeconomic status, and baseline levels of outcomes. We will test for mediation of effects through hypothesized pathways including self-regulation/executive functions as mediators between PA and learning (Howie and Pate, 2012), and socioemotional characteristics, physical fitness, motor skills, and adiposity as mediators between PA and cognitive outcomes (Tomporowski et al., 2011). Mediation analyses include the use of SEM. Importantly, because we include three time points, the design allows for testing a full mediation model (Little, 2013).

###### Association analyses

The large sample allows for analyses of distributions and correlates among the included variables in cross-sectional analyses, as well as analyses of development and tracking over time, and analyses of determinants of change in outcomes in longitudinal analyses in the control group. Cross-lagged panel analysis will be used to determine reciprocal relationships among variables over time (Little, 2013). If there are small and non-significant intervention effects, longitudinal analyses will include both groups to increase power of these analyses. In addition to standard regression models, we will use multivariate pattern analysis (Aadland et al., 2018a, b) to explore multivariate association patterns among the included variables. These analyses include associations related to intervention effects performed by associating changes over time (exposure) to group (outcome), to support the interpretation of the study effects. Multivariate pattern analysis will be performed in Sirius v.11 (Pattern Recognition Systems AS, Bergen, Norway); otherwise, we will use the same statistical models (LMM and SEM) and software, as specified above.

#### The Preschool Level

The individual interviews and focus groups will be recorded, transcribed verbatim, and imported with field notes of observations and documents from each preschool into NVivo-11 for data management. The data will be thematically analyzed following the procedures proposed by Braun and Clarke (2006). The Normalization Process Theory will provide a conceptual framework to understand and evaluate how staff make sense of the intervention (coherence), engage with the professional development (cognitive participation), integrate PA practices in their preschool’s daily practices (collective action), and evaluate the effects of improved quality and quantity of PA practices into their daily operations (reflexive monitoring). Data will be analyzed subsequently and arranged into, within, and between preschool data displays to explore convergences and divergences between the case study preschools. The preliminary displays will also help to identify potential themes and gaps in the data that need to be explored during the second and third round of data collection.

Staff PA will be analyzed using similar statistical approaches as described for child-level outcomes above (i.e., effects will be determined using a between-group comparison). For other data, analyses will be restricted to examination of main effects of time because we only include repeated measurements for the intervention group. Preschool level data will be used as a basis for per-protocol efficacy analyses of individual level outcomes. For these analyses, preschool level data will be used to decide which preschools should be included using appropriate cut points for acceptable participation and engagement in the intervention activities and/or by including these data as explanatory variables in regression models. In association analyses between individual and preschool level data, data will be combined using preschool averages.

## Discussion

We believe professional development of staff and a whole-child approach that integrates physical activity with challenging motor tasks, cognitively engaging play, and learning activities in the early childhood education and care setting, may provide a feasible venue to favorably affect different aspects of both physical and cognitive development in young children. Thus, the ACTNOW intervention model might provide an efficient, acceptable, and sustainable way to build human capital (Bailey et al., 2013) and provide an early solution to lifelong public health and developmental challenges. Of importance for the dissemination and scaling, our intervention model is framed within an education module that may be included in early childhood education and care educational programs. We regard this feature a significant strength of our approach. In addition to this feature, we regard the co-creation of the intervention in partnership with staff, a vital premise for creating sustainable effects that have the potential to extend beyond previous effectiveness studies (Finch et al., 2016; Wick et al., 2017). In this way, we lay the foundation for the intervention’s unique operationalization within each preschool’s context, which value and foster the preschool staff’s autonomy and ownership.

While the flexible and holistic nature of this effectiveness trial has the advantage of testing intervention effects in a real-word setting, the effects of the multifaceted intervention will be compared with those of “business as usual.” The absence of another experimental group with different intervention characteristics limits our ability to improve the understanding of which specific features of the intervention are responsible for any observed effects. However, the inclusion of outcome measures supposed to capture child development across all four core components of PA could be partly applied to elucidate such effects. Most importantly, though, the aim of the study is not to provide evidence in support of specific hypothesis of the PA-cognition relation (Best, 2010; Meeusen et al., 2018), but to test a pragmatic program of enriched and meaningful PA that with minimal adjustment could be adopted by preschools and improve children’s everyday opportunities to take part in developmentally appropriate physically active play.

While randomized trials are highly valued and regarded as the gold-standard approach for making causal inferences, conducting large-scale cluster RCTs with comprehensive and demanding interventions in educational settings poses substantial challenges. It requires acceptance, motivation, and substantial support from preschool owners, directors, and staff, which builds on mutual respect and trust, and a solution-oriented spirit, between researchers and the practice field. To this end, we have no guarantee that ACTNOW will succeed with regard to its implementation and with regard to producing positive child developmental outcomes. These challenges, though, provide a strong rationale for including a comprehensive process evaluation to capture how, why, and for whom the intervention may or may not work (Bonell et al., 2012). Thus, irrespective of the success of the study, we aim to improve knowledge on contextual mechanisms underpinning how the intervention interacts with and produces various organizational outcomes to increase probability of success of future interventions and scaling.

## Dissemination

ACTNOW has the potential to reach a large number of children, parents, teachers, preschools, and authorities, and thereby may serve as a vehicle to positively influence policy in the preschool sector. Beyond sharing knowledge in scientific channels and in popular science, dissemination on local, regional, and national levels will have several components. First, if the professional development/continuing education is successful, we will be able to offer it as part of the university’s educational provision in the long-term. Second, we will use established meeting arenas to inform and stimulate an ongoing dialogue with municipalities and other local, regional, and national stakeholders. Collectively, this means that the distance from testing to widespread dissemination is short, which will promote scaling of the intervention in a real-world context.

## Ethics Statement

The study is approved by the Institutional Ethics Committee and the Norwegian Centre for Research Data (reference number 248220). ACTNOW will study children, a vulnerable group that is unable to give valid consent. We will obtain written informed consent from each child’s parent/guardian prior to testing. Children will be informed about the study procedures on their premises. We will perform all child testing in close collaboration with preschool staff to provide a safe environment for children. Testing will be terminated if a child expresses discomfort. Similar to children/parents, staff will provide written informed consent, specifically tailored to the research activities they participate in, prior to all testing. Procedures and methods will conform to the ethical guidelines defined by the World Medical Association’s Declaration of Helsinki and its subsequent revisions (World Medical Association [WMA], 2017).

## Author Contributions

EA and KA drafted the study protocol. All authors contributed to the design of the study, provided input on the protocol, and approved the final version for publication.

## Conflict of Interest

The authors declare that the research was conducted in the absence of any commercial or financial relationships that could be construed as a potential conflict of interest.

## Footnotes

1 https://clinicaltrials.gov/ct2/show/NCT04048967?term=actnow&rank=1
